# Double-Masked, Vehicle-Controlled, Randomized, Phase II Study of the Ocular Hypotensive Activity and Safety of VVN539 Ophthalmic Solution

**DOI:** 10.1016/j.xops.2023.100426

**Published:** 2023-11-07

**Authors:** David Wirta, Xiao-Yan Li, Wang Shen, Caroline Lu, Gary D. Novack, VVN539-CS201 Study Group, William Christie, Paul J. Hartman, Lawrence Tafoya, Navin Tekwani, St Louis, David Wirta

**Affiliations:** 1 Clinical Research, Eye Research Foundation, Newport Beach, California, USA; 2 Research & Development, VivaVision Biotech, Inc; 3 Research & Development, PharmaLogic Development, Inc., San Rafael, California; 4 Department of Ophthalmology & Vision Science, University of California School of Medicine, Davis, Sacramento, California

**Keywords:** Glaucoma, Intraocular pressure, Ocular hypertension, Rho-kinase inhibitor, VVN539

## Abstract

**Purpose:**

To assess safety and ocular hypotensive efficacy of VVN539 ophthalmic solution in a first-in-human study.

**Design:**

Multicenter, double-masked, randomized, vehicle-controlled, dose-response, parallel-comparison study.

**Participants:**

Sixty-eight subjects with ocular hypertension (OHT) or open-angle glaucoma enrolled at 5 private practices.

**Methods:**

After washout of ocular hypotensive medications as required, the subjects were randomized to receive either VVN539 ophthalmic solution 0.02%, 0.04%, or vehicle once-daily (QD) in the morning (5 days), once-daily in the evening (6 days) and then twice-daily (6 days).

**Main Outcome Measures:**

Comparison of VVNM539 to its vehicle in mean intraocular pressure (IOP) at each diurnal time point (8:00am, 10:00am, and 4:00pm) at visit 4 (day 7), visit 5 (day 14), and visit 6 (day 21).

**Results:**

Mean IOP decreased throughout dosing in the active groups to between 18 and 20 mmHg in both active groups, to between 22 to 23 mmHg in the vehicle group. VVN539 0.04% was statistically superior to vehicle at all 9 diurnal time points (QD AM, QD PM, and twice daily, P ≤ 0.0109). VVN539 0.02% was statistically superior to vehicle at only 6 of 9 diurnal time points (selected QD times and twice daily). The most common ocular treatment-emergent adverse event was conjunctival hyperemia (11 [47.8%], 10 [4.5%], and 1 [4.3%]), followed by ocular hyperemia (3 [13.0%], 5 [22.7%] and 0), respectively. There were no clinically significant changes of note in visual acuity, biomicroscopy, dilated ophthalmoscopy, blood chemistry, hematology, or cardiovascular measures.

**Conclusions:**

In conclusion, the results of this initial phase II study indicate that VVN539 ophthalmic solution showed clinically and statistically significant ocular hypertensive activity and was relatively well tolerated for the treatment of subjects with primary open-angle glaucoma or OHT. Additional studies will be required for a more complete evaluation of the utility of VVN539 ophthalmic solution.

**Financial Disclosure(s):**

Proprietary or commercial disclosure may be found in the Footnotes and Disclosures at the end of this article.

Glaucoma is a major public health issue worldwide, threatening visual function for tens of millions of patients. The disease is treated by lowering intraocular pressure (IOP) either by medical, laser, or surgical means.^1^, ^2^, ^3^, ^4^ With respect to medical treatment, there are several classes of therapy, and within most classes, several molecules are available.^5^ However, even with various treatment options, some patients with glaucoma continue to experience progressive loss of visual function. Thus, new therapies are being investigated.

The class that has most recently entered our armamentarium is the Rho-kinase inhibitors (RKIs).^6^ Netarsudil is available in the United States (US) and Europe, and ripasudil is available in Japan. Additionally, there is a fixed-dose combination of netarsudil and latanoprost available in the US and Europe, and a fixed-dose combination of ripasudil and brimonidine under evaluation in Japan.^7^ More recently, a phase I/II study on a newer agent, H1337, has been reported.^8^ Although a welcome new class of agent, existing RKIs are less than ideal on the magnitude of ocular hypotensive efficacy, and a large proportion of patients experience undesirable conjunctival hyperemia.

VivaVision is developing VVN539, an RKI with nanomolar potency. Upon contact with tissue, it releases nitric oxide (NO) from the nitrate functional group and is metabolized to VIP-5156, a Rho-associated protein kinase (ROCK) inhibitor with subnanomolar potency.^9^ The release of NO from VVN539 is a characteristic like latanoprostene bunod (approved in the US in 2017 and in other countries subsequently).^10^ It has been demonstrated that NO alone can lower the IOP by 10% to 20% (2–4 mmHg) by increasing the outflow facility of aqueous humor.^11^

Nitric oxide released by organic nitrates such as VVN539 stimulates soluble guanylate cyclase, leading to an increase of cyclic Guanosine 3',5'-cyclic monophosphate (cGMP) in trabecular meshwork cells.^12^ This leads to the relaxation of trabecular meshwork, a smooth muscle-like tissue. In addition, NO can also alter calcium-dependent potassium channel conductance, which leads to channel membrane activation and hyperpolarization with lower calcium ions resulting in vascular smooth muscle relaxation.^13^ The IOP lowering mechanisms of action by NO is different from the IOP lowering mechanism of action of RKI.

We hypothesized that there may be synergy or additivity of NO-releasing capacity and RKI. Thus, we conducted a vehicle-controlled, double-masked study of VVN539 in patients with open-angle glaucoma and ocular hypertension (OHT). To assess both safety and ocular hypotensive efficacy of VVN539 in this first-in-human study, evaluating both frequency- and dose-response, we utilized a design previously used in a first-in-human evaluation of the RKI AR-12286 (a predecessor molecule to netarsudil). This is an efficient design for pilot evaluation of not only ocular safety but also ocular hypotensive efficacy to various concentrations and dosing frequency regimens.^14^

## Methods

### Study Design

This was a phase II, multicenter, double-masked, randomized, vehicle-controlled, dose-response, parallel-comparison study to assess the safety and ocular hypotensive efficacy of VVN539 ophthalmic solution in subjects with primary open-angle glaucoma (POAG) or OHT. The study consisted of 6 visits: visit 1 (screening and a washout period of up to 35 days); visit 2 (baseline/randomization), visit 3 (day 1; start treatment), visit 4 (day 7), visit 5 (day 14), and visit 6 (day 21; end of study).

This study was conducted at 5 private practice sites in the US under an Investigational New Drug exemption in accordance with Good Clinical Practice as required by the US Food and Drug Administration regulations. The study was approved by an Institutional Review Board, adhered to the Declaration of Helsinki, and all subjects provided written informed consent before enrollment in the study. This study was registered on clinicaltrials.gov as NCT05451329.

A screening examination was conducted which included a complete eye examination (biomicroscopy, IOP, cup-to-disc ratio, dilated ophthalmoscopy, and (either at that visit or within the previous 3 months): pachymetry, gonioscopy, visual fields (automated threshold visual field), and OCT. Hyperemia was scored on a 0 (none) to 3 (severe) scale using the investigator’s standard of care for illumination. Gonioscopy was scored using the Shaffer system.^15^ Only individuals who demonstrated their ability to instill artificial tear eyedrops in the office to the staff^16^ were enrolled in the study. Qualified individuals using topical ocular hypotensive therapy underwent a washout (prostaglandins, β-adrenoceptor antagonists, kinase inhibitors [4 weeks], adrenergic agonists [2 weeks], muscarinic agonists, and carbonic anhydrase inhibitors [5 days]). After the washout period (if applicable), baseline IOP was taken at 08:00am, 10:00am, and 4:00pm (visit 2, day 1). Subjects meeting all inclusion/exclusion criteria were randomized to 1 of 3 dosing arms in a 1:1:1 ratio: VVN539 at concentrations of 0.02%, or 0.04%, or vehicle (control). Subjects were then instructed to self-administer the investigational product in both eyes in the morning (07:00am to 09:00am) for 5 days. The last once-a-day (QD) morning dose was administered in the clinic during visit 4 (day 7) after the 8:00am IOP measurement was taken. Starting the day after visit 4 (day 7), subjects were told to self-administer the investigational product in the evening (QD evening, 07:00pm to 9:00pm) for 6 days. The last QD evening dose was administered in the evening during visit 5 (day 14) after the last IOP measurement had been taken. Starting the day after visit 5 (day 14), subjects were told to self-administer the investigational product twice-daily for 6 days. The last morning dose (twice daily) was administered in the clinic during visit 6 (day 21) after the 8:00 AM IOP measurement was taken; there was no twice daily evening dose on the final day of study treatment. At the end of visit 6 (day 21), subjects resumed standard of care treatment. Central corneal thickness was assessed by pachymetry predosing and at the end of the study. Blood samples were taken predosing and at the end of the study for clinical chemistry and hematology. Heart rate and blood pressure were taken throughout the study. Adverse events (AEs) were coded using the Medical Dictionary for Regulatory Activities (MedDRA, version 24.1) system, a standard for Good Clinical Practices.

### Subject Eligibility

This study was conducted in subjects ≥ 18 years of age who were diagnosed with POAG or OHT in both eyes and were either untreated for these conditions or had the conditions well controlled with a stable regimen of ≤ 2 ocular hypotensive medications (fixed-dose combinations counted as 2 medications) within 30 days before visit 1 (screening). Also required was unmedicated IOP of ≥ 22 mmHg and ≤ 36 mmHg in the study eye, with no > 5 mmHg intereye difference at 08:00am and 10:00am at visit 2 (baseline/randomization), corrected visual acuity in each eye + 1.0 logarithm of the minimum angle of resolution (Snellen equivalent to 20/200) or better by ETDRS in each eye, and central corneal thickness of ≥ 400 and ≤ 620 μm in each eye. Excluded were individuals with an intraocular implant for IOP treatment, glaucoma filtering surgery, placement or removal of minimally invasive glaucoma implant in the study eye, a history of laser IOP lowering surgery within 6 months, laser peripheral iridotomy for narrow-angle within 3 months, clinically significant ocular disease in either eye (e.g., corneal edema, uveitis, severe keratoconjunctivitis sicca), had pseudoexfoliative, pigmentary, congenital, developmental or secondary glaucoma (e.g., neovascular, uveitic, pigmentary, lens-induced, corticosteroid-induced, trauma-induced, or glaucoma associated with increased episcleral venous pressure) in either eye and closed angle glaucoma as judged by gonioscopy. Also excluded were individuals with severe glaucoma as judged by imaging or visual fields, and women of childbearing potential who were pregnant, nursing, planning a pregnancy, or not using a medically acceptable form of birth control.

### Study Drugs

VVN539 ophthalmic solution is a 0.02% benzalkonium chloride-preserved, isotonic, sterile ophthalmic solution buffered at pH 4.5 to 5.7 and was supplied in 2 concentrations (w/v: 0.02% and 0.04%). The vehicle was identical in formulation to the VVN539 study drug product but without VVN539.

### IOP Assessment

Intraocular pressure was taken and read by Goldmann applanation tonometry by a 2-person method. Two consecutive IOP measurements were taken at each IOP time point. The applanation probe was withdrawn between measurements. The average of the 2 measurements was used for analysis. If the 2 measurements differed by > 4 mmHg, a third measurement was taken, and the median value was used for analysis.^17^

### Statistics

The primary objective was to evaluate the ocular hypotensive efficacy of 2 concentrations of VVN539 ophthalmic solution (0.04% and 0.02%) in subjects with POAG or OHT. The secondary objective was to evaluate the ocular and systemic safety of the 2 concentrations of VVN539 ophthalmic solution in the subject population. The primary efficacy analysis was conducted on the full analysis set, defined as all subjects who were randomized to treatment. The primary analysis was conducted using only observed data and assuming missing at random using a longitudinal model (mixed model repeated measures). Data from days 7, 14, and 21 were analyzed using mixed model repeated measures with an unstructured covariance assumed for each treatment with treatment, visit, and visit by treatment interaction as fixed effects, baseline measurement as a covariate, and a random effect for site (SAS version 9.4, SAS Institute, Cary, NC).

The primary efficacy analysis was a comparison between the VVN539, 0.04%, and vehicle groups in mean IOP at each diurnal time point (8:00am, 10:00am, and 4:00pm) at visit 4 (day 7), visit 5 (day 14), and visit 6 (day 21) using data from the study eye. If observed differences for all 9 diurnal time points were statistically significant at the α = 0.05 level, VVN539, 0.04% was to be declared superior to vehicle and testing was to proceed to a comparison between the VVN539, 0.02%, and vehicle groups. To claim superiority, all 9 diurnal time points had to show statistical significance; therefore, no adjustment to the individual confidence intervals was required. Also calculated was the mean diurnal IOP, the average of all 3 IOP measurements on each study day.

A priori, with a sample size in each group of 20, the study had 80% power to detect a difference of 3.0 mmHg between a VVN539 dose compared with vehicle at each diurnal time point (8:00am, 10:00am, and 4:00pm) assuming a common standard deviation of 3.3 mmHg, α = 0.05 (2-tailed). Probability testing was conducted in a hierarchy (0.04%, then 0.02%) to protect the alpha level. There was no correction for multiplicity for multiple time points or comparisons for high or low VVN539 doses.

The study eye was defined as the qualifying eye with higher IOP at 8:00am at visit 2 (baseline/randomization). If both eyes were qualified and had the same IOP, the right eye was designated as the study eye. Efficacy analyses focused only on the study eye, although supportive analyses were presented by the nonstudy eye, irrespective of study qualification. Ophthalmic safety analyses were presented for both eyes.

## Results

### Disposition, Demographics and Baseline Characteristics

Enrolled in the study were 68 subjects. The mean age of the study population was 66.3 years (range: 21–84 years). Overall, the majority of subjects (45/68 [66.2%] subjects) were ≥ 65 years of age. The proportion of male and female subjects was comparable (35/68 [51.5%] subjects were male; 33/68 [48.5%] subjects were female). The most common race was White (60/68 [88.2%] subjects), followed by Black (4/68 [5.9%] subjects); Asian (2/68 [2.9%] subjects) and Unknown (2/68 [2.9%] subjects) composed the remaining subjects. Most subjects (57/68 [83.8%] subjects) were non-Hispanic or Latino. Mean IOP at baseline in the study eye was similar between groups at each diurnal time point (8:00am, 10:00am, and 4:00pm) (range: 24.8–25.4, 24.1–25.0, and 22.4–23.0, respectively, Table 1). Predosing automated threshold visual fields were in the mild glaucomatous range (mean defect of –0.6 +/– 0.3 decibels [dB], mean ± standard error of the mean [SEM], range of + 1.8 to 6.9).

**Table 1:** Demographics and Baseline Characteristics

	VVN539, 0.02% (N = 23) n (%)	VVN539, 0.04% (N = 22) n (%)	Vehicle (N = 23) n (%)	Overall (N = 68) n (%)
Age (yrs)				
Mean (SD)	69.1 (9.0)	65.1 (13.4)	64.7 (16.4)	66.3 (13.2)
Min; Max	47; 84	21; 84	25; 82	21; 84
Age categories				
< 18 yrs	0 (0)	0 (0)	0 (0)	0 (0)
18 to ≤ 64 yrs	5 (21.7)	9 (40.9)	9 (39.1)	23 (33.8)
≥ 65 yrs	18 (78.3)	13 (59.1)	14 (60.9)	45 (66.2)
Sex				
Male	11 (47.8)	13 (59.1)	11 (47.8)	35 (51.5)
Female	12 (52.2)	9 (40.9)	12 (52.2)	33 (48.5)
Race				
White	18 (78.3)	19 (86.4)	23 (100.0)	60 (88.2)
Black	1 (4.3)	3 (13.6)	0 (0)	4 (5.9)
Asian	2 (8.7)	0 (0)	0 (0)	2 (2.9)
Unknown	2 (8.7)	0 (0)	0 (0)	2 (2.9)
Multiple	0 (0)	0 (0)	0 (0)	0 (0)
Ethnicity				
Hispanic or Latino	6 (26.1)	2 (9.1)	3 (13.0)	11 (16.2)
Non-Hispanic or Latino	17 (73.9)	20 (90.9)	20 (87.0)	57 (83.8)
Baseline IOP (mmHg), 8:00am				
Mean (SD)	25.2 (2.3)	24.8 (2.1)	25.4 (2.5)	25.1 (2.3)
Min; Max	22; 30	22; 29	22; 32	22; 32
Baseline IOP (mmHg), 10:00am				
Mean (SD)	25.0 (2.5)	24.1 (2.1)	24.3 (2.1)	24.5 (2.2)
Min; Max	22; 30	22; 31	22; 29	22; 31
Baseline IOP (mmHg), 4:00pm				
Mean (SD)	22.4 (3.1)	22.8 (2.3)	23.0 (1.7)	22.7 (2.4)
Min; Max	16; 28	19; 29	20; 26	16; 29
Baseline IOP (mmHg), diurnal				
Mean (SD)	24.2 (2.3)	23.9 (1.6)	24.2 (1.8)	24.1 (1.9)
Min; Max	21; 29	22; 27	22; 28	21; 29
Normal nerve fiber layer thickness				
Yes	23 (100.0)	21 (95.5)	22 (95.7)	66 (97.1)
No	0 (0)	1 (4.5)	1 (4.3)	2 (2.9)
Visual field				
Normal	21 (91.3)	19 (86.4)	22 (95.7)	62 (91.2)
Abnormal	2 (8.7)	3 (13.6)	1 (4.3)	6 (8.8)
Central corneal thickness (μm)				
Mean (SD)	551.28 (34.82)	567.67 (25.57)	571.41 (29.21)	563.39 (31.01)
Min; Max	486.0; 620.0	523.0; 617.0	515.0; 620.0	486.0; 620.0
Gonioscopy				
0 (Closed)	0 (0)	0 (0)	0 (0)	0 (0)
I (10–15 degree)	0 (0)	0 (0)	0 (0)	0 (0)
II (15–25 degree)	0 (0)	0 (0)	0 (0)	0 (0)
III (25–35 degree)	13 (56.5)	11 (50.0)	10 (43.5)	34 (50.0)
IV (> 35 degree)	10 (43.5)	11 (50.0)	13 (56.5)	34 (50.0)
Study eye				
OD	17 (73.9)	10 (45.5)	11 (47.8)	38 (55.9)
OS	6 (26.1)	12 (54.5)	12 (52.2)	30 (44.1)
Iris color				
Brown	17 (73.9)	8 (36.4)	11 (47.8)	36 (52.9)
Blue	2 (8.7)	7 (31.8)	11 (47.8)	20 (29.4)
Hazel	2 (8.7)	5 (22.7)	0 (0)	7 (10.3)
Green	2 (8.7)	1 (4.5)	1 (4.3)	4 (5.9)
Other	0 (0)	1 (4.5)	0 (0)	1 (1.5)

Shaffer grades that were not ≥ III were exclusionary.

Percentages were based on the number of nonmissing observations in each group and overall.

Central corneal thickness was the average of 3 measurements.

Percentages were based on the number of nonmissing observations in each group and overall.

Subjects were in a particular race category if it was the only 1 selected; otherwise, they were counted in multiple.

Glaucoma diagnosis adds up to > 100% due to different diagnoses in eyes within a subject.

IOP = intraocular pressure; OD = oculus dexter (right eye); OS = oculus sinister (left eye); SD = standard deviation.

**Figure 1:**
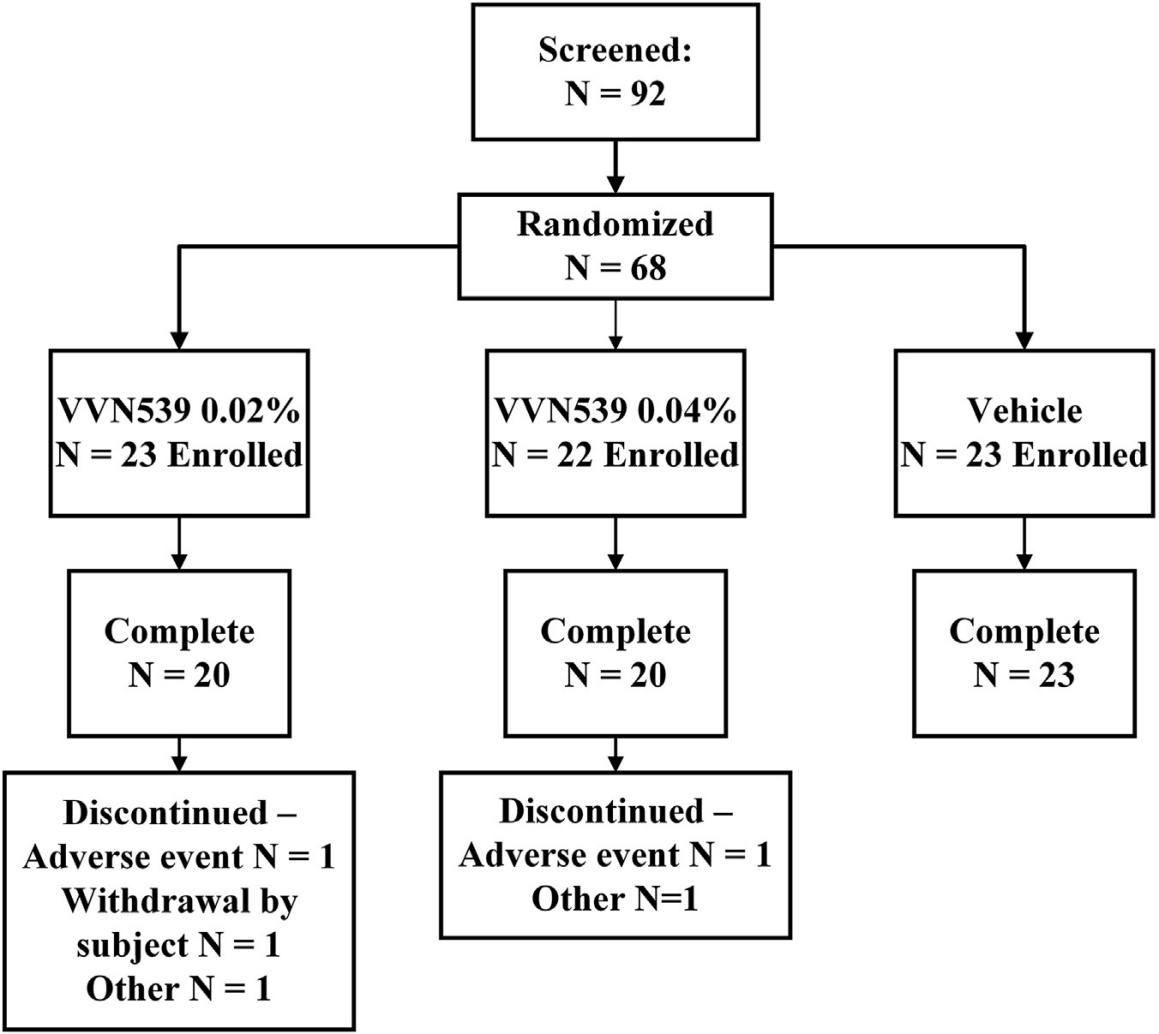
Flow chart.

Most (63/68 [92.6%] subjects) of the subjects randomized completed the study. Five subjects did not complete the study: (3 in the VVN539 0.02% group, and 2 in the VVN539 0.04% group). Of these 5 subjects, 1 each was discontinued for the AE of conjunctival hyperemia (Fig 1). One subject in the vehicle group had a rescue medication added. There was 1 major protocol deviation—1 subject in the 0.02% treatment group had a cup-disc ratio of 0.7, exceeding the protocol specification of ≤ 0.6.

### Ocular Hypotensive Efficacy

**Figure 2:**
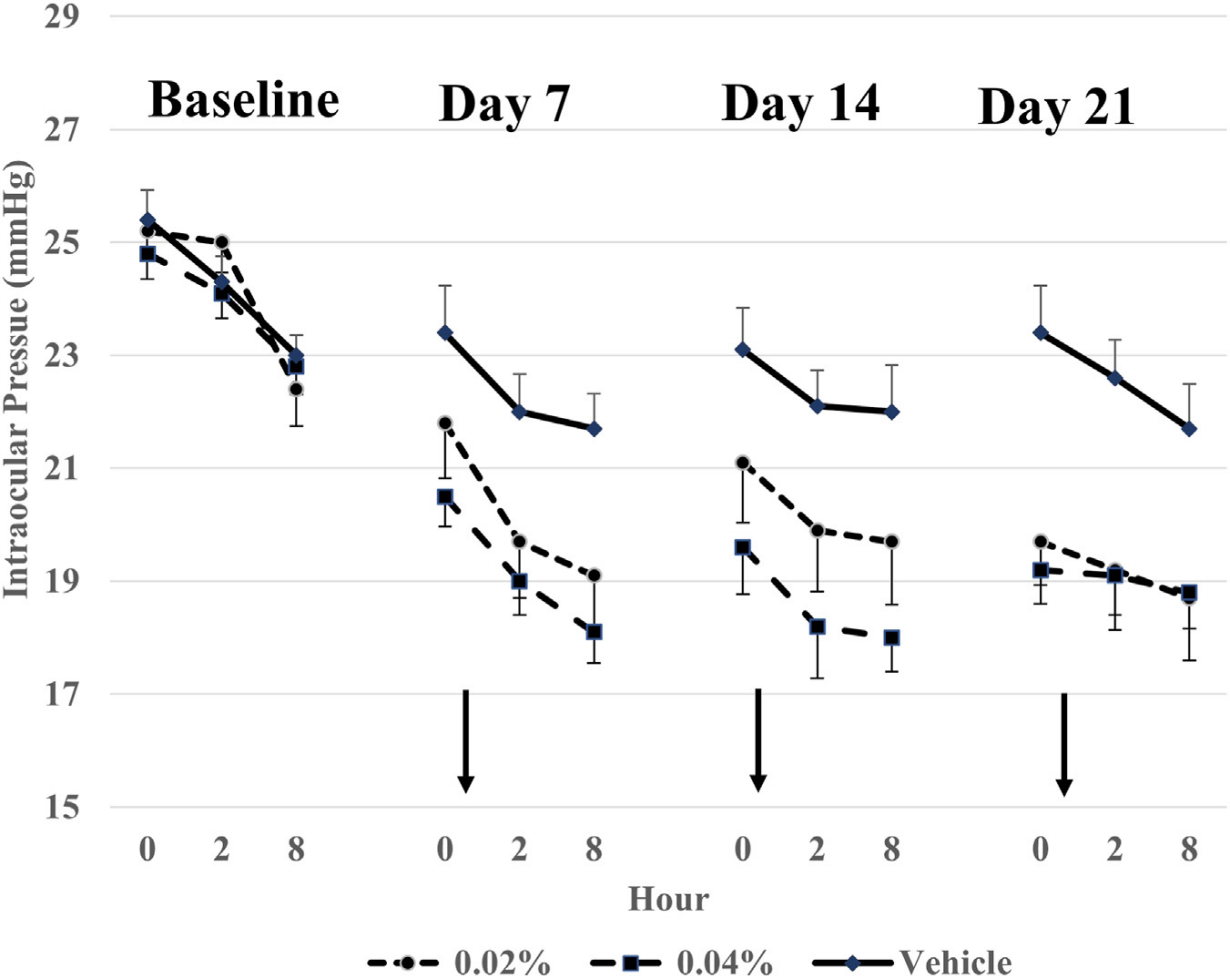
Intraocular pressure: mean (± standard error of the mean) (Intent to Treat population, mmHg). Each active group was statistically significantly different from vehicle at both day 14 and day 28 (*P* < 0.001).

Mean IOP decreased throughout dosing in the active groups to between 18 and 20 mmHg in both active groups, to between 22 to 23 mmHg in the vehicle group (Fig 2). VVN539 0.04% was statistically superior to vehicle at all 9 diurnal time points (QD AM, QD PM, and twice daily, *P* ≤ 0.0109). VVN539 0.02% was statistically superior to vehicle at only 6 of 9 diurnal time points (selected QD times and twice daily). This within-group change from baseline was statistically significant for all 9 diurnal time points in both active groups and at 7 of 9 diurnal time points in the vehicle group (Table 2). The decrease in mean IOP was seen throughout dosing in the active groups of 4 to 6 mmHg and in the vehicle group of 1 to 2 mmHg (Table 3). Mean diurnal IOP showed a decrease in the active groups of 4 to 5 mmHg and in the vehicle group of 1 to 2 mmHg. Both active groups (VVN539, 0.04%, and VVN539, 0.02%) achieved statistically significant superiority to the vehicle group at all visits (*P* ≤ 0.0004 and *P* ≤ 0.0152, respectively, Table 4).

**Table 2:** IOP: Mean Difference and *P* Value Comparisons for Change from Baseline IOP (mmHg) at Each Diurnal Time Point Between Active Treatment Groups (VVN539) and Vehicle by Visit and Time Point, Study Eye

	8:00am		10:00am		4:00pm	
	VVN539, 0.02% (N = 23)	VVN539, 0.04% (N = 22)	VVN539, 0.02% (N = 23)	VVN539, 0.04% (N = 22)	VVN539, 0.02% (N = 23)	VVN539, 0.04% (N = 22)
Day 7, QD AM						
LS mean difference	–1.69	–2.11	–2.94	–2.68	–2.24	–3.36
(95% CI)	(–3.92, 0.54)	(–3.70, –0.51)	(–5.30, –0.59)	(–4.37, –0.99)	(–4.12, –0.37)	(–4.73, –1.98)
P value	0.1318	**0.0109**	**0.0159**	**0.0028**	**0.0206**	**< 0.0001**
Day 14, QD PM						
LS mean difference	–2.08	–2.80	–2.87	–3.56	–2.00	–3.81
(95% CI)	(–4.20, 0.03)	(–4.75, –0.85)	(–5.06, –0.68)	(–5.33, –1.80)	(–4.61, 0.60)	(–5.77, –1.86)
P value	0.0536	**0.0062**	**0.0121**	**0.0003**	0.1273	**0.0003**
Day 21, twice daily						
LS mean difference	–3.83	–3.31	–4.05	–2.94	–2.64	–2.56
(95% CI)	(–5.56, –2.09)	(–5.03, –1.59)	(–6.14, –1.96)	(–4.85, –1.03)	(–5.06, –0.22)	(–4.35, –0.76)
P value	**< 0.0001**	**0.0004**	**0.0004**	**0.0037**	**0.0333**	**0.0064**

Mean (LS mean), difference, 95% CI, and *P* values were from a repeated measures model with treatment, visit, and visit by treatment interaction as fixed effects, time-matched baseline IOP measurement as a covariate, and a random effect for site. The model assumed an unstructured covariance for each treatment. Models were run separately for 8:00am, 10:00am, and 4:00pm. Statistically significant primary end point results are noted in bold text.

AM = morning; CI = confidence interval; IOP = intraocular pressure; LS mean = least squares mean; PM = evening; QD = once-daily; SD = standard deviation.

**Table 3:** Mean (± SD) Change from Baseline Comparison in Intraocular Pressure (mmHg) at Each Diurnal Time Point by Visit and Time Point, Study Eye

	8:00am			10:00am			4:00pm		
	VVN539, 0.02% (N = 22)	VVN539, 0.04% (N = 23)	Vehicle (N = 23)	VVN539, 0.02% (N = 22)	VVN539, 0.04% (N = 23)	Vehicle (N = 23)	VVN539, 0.02% (N = 22)	VVN539, 0.04% (N = 23)	Vehicle (N = 23)
Day 7, QD AM	–3.6 (4.1)∗	–4.1 (2.7)∗	–2.0 (2.8)∗	–5.3 (4.2)∗	–5.0 (3.0)∗	–2.3 (3.0)∗	–3.3 (3.5)∗	–4.6 (2.6)∗	–1.3 (2.2)
Day 14, QD PM	–4.3 (4.3)∗	–5.0 (4.0)∗	–2.2 (2.7)∗	–5.1 (4.2)∗	–5.8 (3.6)∗	–2.1 (2.3)∗	–2.7 (4.6)∗	–4.8 (3.3)∗	–0.9 (3.5)
Day 21, twice daily	–5.7 (2.7)∗	–5.4 (2.8)∗	–2.0 (3.3)∗	–5.7 (3.9)∗	–5.0 (3.7)∗	–1.5 (2.4)∗	–3.7 (4.3)∗	–3.9 (2.8)∗	–1.2 (3.4)

AM = morning; PM = evening; QD = once-daily; SD = standard deviation.

∗ *P* ≤ 0.05 for within-group comparison.

**Table 4:** Mean (± SD) Diurnal Intraocular Pressure (mmHg): Comparison Between Active Treatment Groups (VVN539) and Vehicle, Study Eye and within-Group

	VVN539, 0.04% (N = 22)	VVN539, 0.02% (N = 23)	Vehicle (N = 23)
Baseline			
Mean (SD)	23.9 (1.6)	24.2 (2.3)	24.2 (1.8)
Day 7, QD AM			
Mean (SD)	–4.5 (2.3)∗	–4.0 (3.3)∗	–1.7 (2.1)∗
LS mean (StdErr)	–4.42 (0.64)	–4.09 (0.86)	–1.77 (0.59)
LS mean difference	–2.65	–2.31	–
(95% CI)	(–3.93, –1.37)	(–4.07, –0.55)	–
P value	0.0002	0.0117	–
Day 14, QD PM			
Mean (SD)	–5.1 (3.2)∗	–3.9 (3.8)∗	–1.5 (2.3)∗
LS mean (StdErr)	–5.04 (0.79)	–3.99 (0.91)	–1.56 (0.63)
LS mean difference	–3.49	–2.43	–
(95% CI)	(–5.14, –1.83)	(–4.36, –0.50)	–
P value	0.0001	0.0152	–
Day 21, twice daily			
Mean (SD)	–4.8 (2.6)∗	–5.0 (2.9)∗	–1.7 (2.6)∗
LS mean (StdErr)	–4.59 (0.69)	–5.04 (0.76)	–1.73 (0.65)
LS mean difference	–2.86	–3.31	–
(95% CI)	(–4.34, –1.37)	(–4.95, –1.67)	–
P value	0.0004	0.0002	–

LS mean, difference, 95% CI, and *P* value were from a repeated measures model with treatment, visit, and visit by treatment interaction as fixed effects, diurnal baseline IOP measurement as a covariate, and a random effect for site. The model assumes an unstructured covariance for each treatment.

Diurnal IOP was the average of 8:00am, 10:00am, and 4:00pm measurements.

AM = morning; CI = confidence interval; IOP = intraocular pressure; LS mean = least squares mean; PM = evening; QD = once-daily; SD = standard deviation; StdErr = standard error.

∗ *P* ≤ 0.05 for within-group comparison.

Results from the per-protocol analysis (in which 1 subject with major protocol deviation and 5 noncompleting subjects were excluded) were similar to the full analysis set analysis.

### Safety

Overall, 29 out of 68 (42.6%) subjects had ≥ 1 ocular treatment-emergent AE in either eye (VVN539 0.02%; 14 [60.9%], VVN539 0.04% [14, 63.6%] and vehicle 1/23 [4.3%]). The most common ocular treatment-emergent AE was conjunctival hyperemia (11 [47.8%], 10 [4.5%], and 1 [4.3%]), followed by ocular hyperemia (3 [13.0%], 5 [22.7%], and 0), respectively. All other ocular treatment-emergent AEs occurred in ≤ 3 (≤ 4.4%) of the 68 subjects (Table 5). We evaluated the potential overlap between the MedDRA terms “conjunctival hyperemia” and “ocular hyperemia.” The total number of subjects with either one or both of these AEs was 14/22 (63.6%), 13/23 (56.5%), and 1/23 (4.3%) in the VVN539, 0.04%, VVN539, 0.02%, and vehicle groups, respectively. One subject lost up to 0.20 logarithm of the minimum angle of resolution in both eyes (OU) (2 lines ETDRS) at days 7, 14, and 21 (AE of “visual acuity reduced”). This same subject experienced “worsening cataract” OU at the final visit. Both AEs were judged unrelated to the study medication by the investigator. No verticillata were observed.

**Table 5:** Treatment-Emergent Ocular Adverse Events by System Organ Class and Preferred Term

System Organ Class Preferred Term	VVN539, 0.02% (N = 23) n (%)	VVN539, 0.04% (N = 22) n (%)	Vehicle (N = 23) n (%)
Subjects with any ocular TEAEs	14 (60.9)	14 (63.6)	1 (4.3)
Eye disorders	14 (60.9)	14 (63.6)	1 (4.3)
Conjunctival hyperemia	11 (47.8)	10 (45.5)	1 (4.3)
Ocular hyperemia	3 (13.0)	5 (22.7)	0 (0)
Eye pain	1 (4.3)	3 (13.6)	0 (0)
Eye irritation	1 (4.3)	2 (9.1)	0 (0)
Vision blurred	1 (4.3)	2 (9.1)	0 (0)
Lacrimation increased	1 (4.3)	1 (4.5)	0 (0)
Blepharitis	0 (0)	1 (4.5)	0 (0)
Cataract	0 (0)	1 (4.5)	0 (0)
Conjunctival hemorrhage	0 (0)	1 (4.5)	0 (0)
Swelling of eyelid	0 (0)	1 (4.5)	0 (0)
Visual acuity reduced	0 (0)	1 (4.5)	0 (0)
Eye pruritus	1 (4.3)	0 (0)	0 (0)
Eyelid bleeding	1 (4.3)	0 (0)	0 (0)
Foreign body sensation in eyes	1 (4.3)	0 (0)	1 (4.3)
General disorders and administration site conditions	1 (4.3)	0 (0)	0 (0)
Instillation site foreign body sensation	1 (4.3)	0 (0)	0 (0)

AE = adverse event; IP = investigational product; MedDRA = Medical Dictionary for Regulatory Activities; TEAE = treatment-emergent adverse event.

TEAE was defined as an AE that started on or after the date of the first dose of IP, up to and including the last date of IP dosing.

Subjects with ≥ 1 AEs within a level of MedDRA were counted only once in that level.

Percentages were based on the number of subjects in each group.

There were a total of 6 nonocular AEs, 2 subjects in each treatment group. For 1 of these reports, headache in the VVN539 0.02% group, the event was judged to be related to study medications (Table 6). There were no serious AEs reported.

**Table 6:** Treatment-Emergent Nonocular Adverse Events by System Organ Class and Preferred Term

System Organ Class Preferred Term	VVN539, 0.02% (N = 23) n (%)	VVN539, 0.04% (N = 22) n (%)	Vehicle (N = 23) n (%)
Subjects with any nonocular TEAEs	2 (9.1)	2 (8.7)	2 (8.7)
General disorders and administration site conditions	0 (0)	0 (0)	1 (4.3)
Peripheral swelling	0 (0)	0 (0)	1 (4.3)
Infections and infestations	1 (4.5)	1 (4.3)	0 (0)
Cellulitis	1 (4.5)	0 (0)	0 (0)
Gastroenteritis staphylococcal	1 (4.5)	0 (0)	0 (0)
Upper respiratory tract infection	0 (0)	1 (4.3)	0 (0)
Investigations	0 (0)	0 (0)	0 (0)
Blood pressure increased	0 (0)	0 (0)	0 (0)
Metabolism and nutrition disorders	0 (0)	0 (0)	1 (4.3)
Diabetes mellitus	0 (0)	0 (0)	1 (4.3)
Nervous system disorders	0 (0)	1 (4.3)	0 (0)
Headache	0 (0)	1 (4.3)	0 (0)

TEAE was defined as an AE that started on or after the date of the first dose of IP, up to and including the last date of IP dosing.

Subjects with ≥ 1 AEs within a level of MedDRA were counted only once in that level.

Percentages were based on the number of subjects in each group.

AE = adverse event; IP = investigational product; MedDRA = Medical Dictionary for Regulatory Activities; TEAE = treatment-emergent adverse event.

There were no clinically significant changes of note in visual acuity, biomicroscopy, dilated ophthalmoscopy, blood chemistry, hematology, or cardiovascular measures.

## Discussion

In this double-masked, vehicle- and dose-controlled, parallel, first-in-human study, topical ocular dosing with VVN539 ophthalmic solution resulted in a clinically and statistically significant decrease in elevated IOP in subjects with POAG or OHT.

The higher concentration of VVN539 ophthalmic solution (0.04%) was statistically superior to its vehicle at all 9 diurnal time points over the course of the 21-day study. In an effort to efficiently evaluate dosing frequency, the study also employed a titration of dosing frequency—QD AM, QD PM, and twice daily. There was little to no increase in efficacy with increased dosing frequency with the 0.04% dose. The 0.02% dose may be more effective given twice daily, especially at 08:00am (before the morning dose). We note that although the treatment assignment was randomized, it was a small study and that subjects in the 0.02% treatment group had more dark-colored irides, higher IOPs, were older, and had thinner corneas. This might have an impact on the apparent dose-response. The magnitude of the decrease from baseline, 4 to 6 mmHg, was numerically similar to published IOP decreases seen with other compounds of this class (i.e., netarsudil and ripasudil).^18^, ^19^, ^20^ However, a true evaluation of the ocular hypotensive efficacy of VVN539 will require a head-to-head comparison with a positive (i.e., approved) product.

The lower concentration of VVN539 ophthalmic solution (0.02%) was statistically superior to its vehicle at 6 out of 9 diurnal time points, including day 21 (twice daily dosing). Consistent with other studies of this type, there was a decrease in IOP in the vehicle group of 1 to 2 mmHg.^18^, ^19^, ^20^, ^21^ Note that technically, due to the sequential testing procedure, *P* values from secondary statistical evaluations (e.g., within-group change from baseline, mean diurnal IOP, etc.), and lack of adjustment for multiplicity, are nominally not usable.

VVN539 ophthalmic solution was relatively well tolerated by subjects on both a QD and a twice daily schedule. Two subjects (1 each from VVN539, 0.04%, and VVN539, 0.02%) withdrew from the study because of an AE of mild ocular hyperemia.

A manual review was conducted of the listings to determine if there was an overlap between the MedDRA terms “conjunctival hyperaemia” and “ocular hyperaemia” within subjects. An overlap was found within subjects. The total number of subjects with either one or both AEs was 13/23 (56.5%), 14/22 (63.6%), and 1/23 (4.3%) in the VVN539, 0.04%, VVN539, 0.02%, and vehicle groups, respectively. All these events were judged mild in severity. Due to the dosing frequency escalation study design, the onset of these AEs was challenging to evaluate. Ocular redness (conjunctival hyperemia and/or ocular hyperemia) is to be expected due to the pharmacology of ROCK inhibitors; similar results appear in the literature.^14^^,^^19^^,^^20^ We did not perform the specialized evaluation of corneal endothelium by specular microscopy in this short study. Typically, this is a US regulatory requirement, performed in later-stage trials of ≥ 3 months duration. In a large controlled study of another molecule of similar pharmacology, no changes were seen in the density of corneal endothelial cells.^22^

In conclusion, the results of this initial phase II study indicate that VVN539 ophthalmic solution showed clinically and statistically significant ocular hypertensive activity and was relatively well tolerated for the treatment of subjects with POAG or OHT. Additional studies will be required for a more complete evaluation of the utility of VVN539 ophthalmic solution.
